# A systematic review of the effect of structured exercise on inflammation and body composition in inflammatory bowel disease

**DOI:** 10.1007/s00384-023-04437-2

**Published:** 2023-05-25

**Authors:** Neasa Mc Gettigan, Kathryn Allen, Reza Saeidi, Aoibhlinn O’ Toole, Karen Boland

**Affiliations:** 1 Department of Medicine, Royal College of Surgeons in Ireland, Dublin, Ireland; 2 Department of Gastroenterology, Beaumont Hospital, Dublin, Ireland

**Keywords:** Inflammatory bowel disease, Ulcerative colitis, Crohn’s disease, Physical activity, Exercise, Sarcopenia

## Abstract

**Purpose:**

Given the substantial risk of treatment failure in inflammatory bowel disease (IBD), adjuvant therapies may play a role in disease management. We aim to carry out a systematic review to examine the effects of structured exercise on the inflammatory response in patients with IBD. Our secondary aim is to examine the effect of structured exercise programmes on body composition given both an increase in visceral obesity and the presence of sarcopenia have deleterious effects on outcomes in IBD.

**Methods:**

A systematic review was carried out following the Methodological Expectations of Cochrane Intervention Reviews (MECIR) manual and the Cochrane Handbook for Systematic Reviews of Interventions. Title/Abstract and MeSH Terms were used to search for relevant studies.

**Results:**

In total, 1516 records were screened for eligibility, and 148 records were reviewed for eligibility, of which 16 were included and a further 7 studies were identified from hand searching references. Four studies included body composition outcomes, and 14 studies reviewed the inflammatory response to exercise.

**Conclusion:**

Further studies of adequate duration are required to include patients with more active disease to demonstrate an inflammatory response to exercise. Body composition measurements including muscle mass and visceral adiposity may play a key role in response to medical therapy in IBD and should be included as exploratory outcomes in future studies. A meta-analysis was not carried out due to the significant heterogeneity amongst studies.

**Supplementary Information:**

The online version contains supplementary material available at 10.1007/s00384-023-04437-2.

## Introduction

Alternative therapies for the treatment of inflammatory bowel disease (IBD) are under-researched and are therefore omitted from current best practice guidelines. Examples of alternative therapies include exercise interventions, dietary supplements, psychotherapies and body-based interventions including acupuncture. IBD remains an incurable condition that encompasses Crohn’s disease (CD), ulcerative colitis (UC) and indeterminate colitis and is characterised by relapsing and remitting clinical episodes [1–3]. Debilitating pathognomonic symptoms include diarrhoea, rectal bleeding, abdominal cramping and weight loss. Other significant symptoms include severe fatigue, arthralgia, low mood and anxiety which are commonly seen in other chronic diseases [4–6].

Disease-modifying medical therapies are continuously under investigation for the treatment of IBD, with novel mechanistic developments emerging regularly. However, biologic therapies remain at the forefront of treatment of moderate to severe IBD since the initial introduction of infliximab in 1997 [7–10]. A relatively high proportion of patients experience primary loss of response to biologic therapy and a further 40% experience secondary loss of response [11–15], most commonly seen in anti-TNF agents. This highlights a potential role for alternative or complementary therapies, including the routine use of personalised exercise programmes. There is an existing knowledge gap in whether there is a role for these strategies and how best to use them within the existing armamentarium. An ECCO topical review on alternative and complementary therapies in IBD, published in 2019, acknowledged that patients increasingly use alternative or complimentary therapies. Reportedly, up to 50% of patients with IBD are estimated to use different forms of complimentary or alternative medicine at some point in their disease course despite a lack of appropriate evidence and guidance [16]. To address this knowledge gap, we believe there is a need for an up-to-date systematic review on the topic of structured exercise programmes and their effect on IBD outcomes, in particular with a focus on the inflammatory response and changes in body composition.

Specific guidelines for exercise in patients with IBD are lacking; however, we note that following a review of published exercise studies, Eckert et al. [17] suggested that patients with IBD should participate in moderate physical activity at least 3 times a week, for at least 30 min a day, incorporating both resistance and aerobic training. This echoes the WHO physical activity recommendations, which advises that patients aged 18–65 years should carry out 150–300 min of moderate intensity or 75–150 min of vigorous-intensity aerobic physical activity a week [18]. The American College of Sports Medicine defines moderate-intensity exercise as ranging from 40 to 60% of maximal exercise capacity (e.g. brisk walking) and high-intensity exercise above 65% of maximal exercise capacity (e.g. jogging) [19]. Low intensity exercises include slow-paced walking, yoga and Qigong. Resistance and aerobic training are two of the most common forms of exercise training. Resistance training has been defined as the use of both static and dynamic muscle actions under tension with an external load (e.g. use of free weights, machine weight training) [20] and aerobic training as any activity that includes the use of large muscle groups that is rhythmic in nature and maintained continuously (e.g. walking, jogging, swimming) [21].

**Fig. 1:**
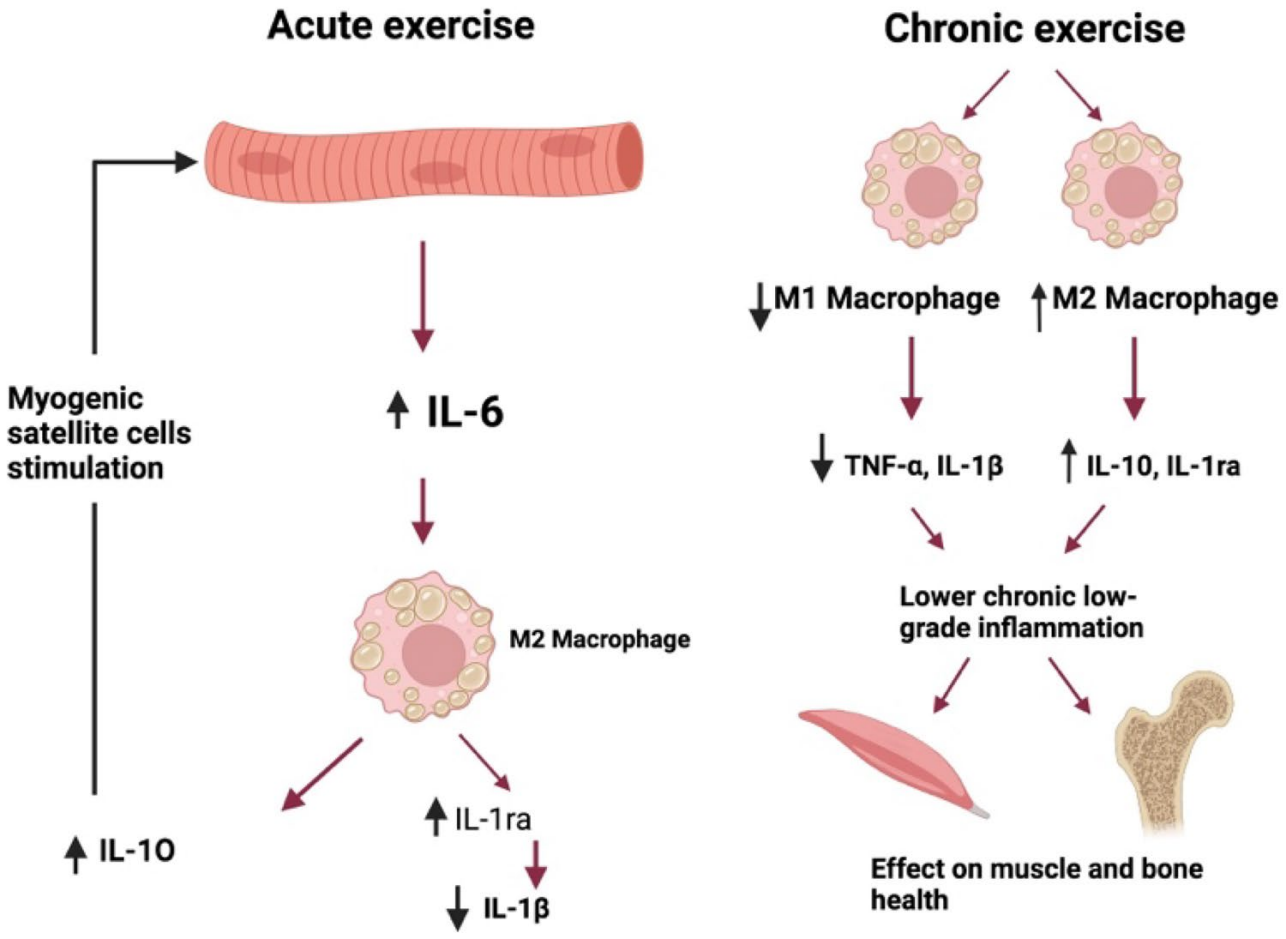
The effects of exercise on pro-inflammatory cytokines. Image created using Biorender

Regular exercise may serve to help regulation of inflammation and promotion of an anti-inflammatory state. Current evidence proposes three possible anti-inflammatory mechanisms of exercise, including (i) the reduction in visceral adiposity, (ii) an increase in production and release of anti-inflammatory cytokines from skeletal muscle and (iii) a reduction in the expression of monocyte and macrophage Toll-like receptors leading to subsequent inhibition of downstream pro-inflammatory cytokine responses [22]. The production of IL-6 (myokine) by contracting skeletal muscle during exercise stimulates the production of the anti-inflammatory cytokines IL-ra and IL-10 which in turn inhibit the production of TNF-alpha, the well-known pro-inflammatory cytokine in IBD (Fig. 1) [23]. Whilst TNF-alpha is a recognised potent trigger of inflammation in IBD, it is also believed to play a role in the metabolic syndrome with data suggesting it impairs insulin signalling, leading to insulin-resistant states [24–26]. Therefore, the IL-6 mediated suppression of TNF-alpha, as a product of regular exercise, offers protection against inflammation and may also lead to the reduction in TNF-alpha-associated insulin resistance [27]. Whilst a reduction in systematic inflammation by regular exercise has been observed in other chronic diseases, this has not yet been proven in IBD [28].

Some data have proposed associations between body composition in relation to muscle mass in addition to adiposity and outcomes in IBD. Visceral obesity defined by an abnormally high deposition of fat around the intra-abdominal organs is associated with lower degree of response to biologics in CD, stricturing disease with higher rates of surgery and also post-operative recurrence [29–35]. Sarcopenia is classically associated with older persons and is characterised by both a loss of skeletal muscle mass, strength and performance [36]. Sarcopenia may be present in over 40% of IBD patients and is associated with disease severity and need for rescue therapy in acute severe colitis in addition to the need for surgery in CD [37, 38]. Patients with IBD often lead more sedentary lifestyles due to their disease burden and debilitating fatigue symptoms which puts them at risk of developing sarcopenia due to the lack of muscle training and increases their risk of developing higher levels of visceral adiposity.

We aim to carry out a systematic review to examine the effects of structured exercise on the inflammatory response in IBD patients. Our secondary aim is to examine the effect of structured exercise programmes on body composition given both an increase in visceral obesity and the presence of sarcopenia have deleterious effects on outcomes in IBD patients. We will also include an overview of the included exercise studies in patients with IBD in our reporting to ensure a comprehensive review and to provide a platform for a well-rounded discussion. The study protocol has been registered with PROSPERO (CRD42022310035).

## Methods

### Study selection

Randomised controlled trials, pilot studies, observational studies, case–control or cross-sectional studies investigating the effects of structured physical activity programmes were included. All forms of exercise were included once they formed part of a formal intervention. Studies that included once-off exercise events were included if they measured relevant outcomes. Studies from 1980 onwards were included (see Table 1). The Methodological Expectations of Cochrane Intervention Reviews (MECIR) manual and the Cochrane Handbook for Systematic Reviews of Interventions were followed during the review [39].

**Table 1:** Study inclusion and exclusion criteria

**Inclusion criteria**	**Exclusion criteria**
Observational, RCTs, Pilot RCTs, case-control, cross-sectional	Review articles, study protocols, surveys, post hoc analysis will not be considered as separate studies
Exercise as an intervention	Measurement of non-specific physical activity (PA) without an intervention, i.e. surveys or questionnaires
Exercise as a treatment of IBD	Exercise/PA relating to the development of IBD
Studies from 1980	Studies prior to 1980
English language unless translated version available	Papers with no translation available not in English
Aerobic, resistance training, yoga, martial arts	Breathing exercises or meditation
IBD patients ≥ 18 years	Paediatric/adolescent studies
Published data/complete results	Incomplete results
Full articles and conference papers	Abstracts only

*RCTs* randomized controlled trials, *IBD* inflammatory bowel disease, *PA* physical activity

### Types of participants

Patients with confirmed ulcerative colitis, Crohn’s disease and indeterminate colitis were considered for inclusion (Table 1). All studies included were carried out in adults > 18 years old; paediatric and adolescent studies were excluded.

### Search methods for identification of studies

#### Electronic searches

The databases Embase, PubMed/MEDLINE and Cochrane Central Register of Controlled trials (CENTRAL) for applicable RCTs were searched from August to December 2021 in accordance with the PRISMA guidelines. Two researchers (NMG and KA) carried out the searches for each database. The Boolean search method was used on PubMed using ‘OR’ and ‘AND’. The PubMed strategy included the use of both MeSH terms and Title/Abstract. All the exercise terms were grouped together with Title/Abstract OR MeSH Terms, followed by the IBD terms and then the exercise terms were combined with the IBD terms using “AND”.

This search was as follows: “exercise”[Title/Abstract] OR “exercise”[MeSH Terms] OR “exercise movement techniques”[MeSH Terms] OR “physical activity”[Title/Abstract] OR “physical and rehabilitation medicine”[MeSH Terms] OR “resistance training”[Title/Abstract] OR “resistance training”[MeSH Terms] OR “resistance exercise”[Title/Abstract] OR “aerobic training”[Title/Abstract] OR “aerobic exercise”[Title/Abstract]) AND (“inflammatory bowel disease”[Title/Abstract] OR “inflammatory bowel diseases”[MeSH Terms] OR “colitis”[MeSH Terms] OR “ulcerative colitis”[Title/Abstract] OR “crohn s disease”[Title/Abstract] OR “crohns disease”[Title/Abstract] OR “IBD”[Title/Abstract].

Our search protocol was agreed upon by both NMG and KA. The protocol was reviewed and approved by a clinical librarian in the Royal College of Surgeons Ireland (RCSI).

#### Searching other resources

To identify other trials, we:

- Examined the reference lists of included studies and review articles for additional citations
- Searched ongoing trials and research registers including ClinicalTrials.gov (www.clinicaltrials.gov) ‘exercise’, ‘physical activity’ and ‘inflammatory bowel disease’ or ‘ulcerative colitis’ or ‘Crohn’s disease’
- Contacted trial authors to identify further published and unpublished trials and asked if they were willing to disclose their unpublished data
- Searched published abstracts from conference proceedings, including the European Crohn’s and Colitis Organisation Congress, Digestive Disease Week and United European Gastroenterology Week
- Searched relevant journals (Journal of Crohn’s and Colitis; Inflammatory Bowel Diseases; Gastroenterology, Alimentary Pharmacology and Therapeutics)

### Data collection and analysis

#### Selection of studies

Two independent review authors (NMG and KA) screened and examined the eligibility of the titles and abstracts identified by the search based on the predetermined inclusion criteria described above. Full‐text papers were sought for all studies meeting the inclusion criteria and were reviewed by two independent review authors (NMG and KA).

Where data was available for IBD participants, trials with a heterogeneous sample of disorders were included. Authors of included studies were contacted regarding information that was unclear or missing. If a disagreement occurred about the selection of a study, arbitration was sought from a third author with content expertise (KB) and a final inclusion decision made by consensus.

#### Data extraction and management

For each included study, NMG and KA independently extracted and documented the relevant data using standardised data extraction forms. All trial authors were contacted to provide additional or unpublished material where relevant. Any disagreements regarding inclusion or exclusion were resolved through arbitration with a senior author (KB) as necessary.

#### Measurement of risk of bias and quality assessment

The GRADE (Grades of Research, Assessment, Development and Evaluation) online assessment tool was used to analyse the certainty of evidence for each outcome (both body composition changes in response to exercise and inflammatory response to exercise). The principal domains reviewed by NM and KA were study design, consistency of effect, imprecision, indirectness, inconsistency and publication bias with the overall certainty graded as high, moderate, low or very low.

The Cochrane ‘Risk of Bias’ (RoB 2.0) tool was used to examine the risk of bias for randomised studies at individual study level for the main outcomes of inflammatory response and body composition changes and the risk of bias was determined as ‘low risk’, ‘some concerns’ and ‘high risk’ [40]. The ‘Risk of Bias In Non-randomised studies of Interventions’ (ROBINS-I) was used for non-randomised studies with each domain classified as ‘low’, ‘moderate’, ‘no information’, ‘serious’ or ‘critical risk’ [41]. The overall judgement was based upon the highest classified risk; therefore, if one domain was high risk, the overall risk of bias was deemed as high.

## Results

### Exercise in IBD

All studies identified for this review are summarised in Table 2. For the purpose of the review, an overview of all included studies is given, and studies have been further stratified to focus on the effect of exercise on inflammatory pathways and body composition in patients with IBD.

**Table 2:** An overview of results of included studies

**Author (s)**	**Study design**	**Patient demographics** **Disease** **Mean age**	**No. of patients**	**Disease activity**	**Type of exercise**	**Duration**	**Outcomes measured**	**Results**
Cronin et al. (2019) [54]	RCT -crossover	UC + CD Mean age = 25 ± 6.5	Total = 20 Intervention = 13 Control = 7	Remission	Aerobic and resistance Moderate intensity	8 weeks	1. Body composition using DEXA 2. Physical fitness VO2max 3. Cytokine analysis 4. Microbiome analysis 5. Anxiety/Mood- HADS, STAI, BDI-II 6. QoL-SF36^®^V2 7. Disease activity scores-CDAI, SCI	1. Improved physical fitness was seen 2. A change in body composition was observed (lean tissue and fat mass) 3. No significant alterations in the α/β-diversity of gut microbiota or metabolic panels 4. No deterioration in disease activity 5. Compliance was high
Ng et al. (2007) [42]	RCT	CD Mean age = Exercise = 40.6 ± 11.7 Control = 37.0 ± 12.7	Total = 32 Intervention = 16 Control = 16	Mild activity or remission	Low intensity walking programme X3 times/week for 30 min	12 weeks	1. QoL 2. Safety	1. Improved QoL with the intervention 2. No deterioration in disease activity
Klare et al. (2015) [55]	RCT	UC + CD Mean age = 41.1 ± 14.1	Total = 30 Intervention = 15 Control = 15	Mild to moderate IBD (CDAI < 220, RI < 11)	Moderate intensity running	10 weeks	1. QoL (IBDQ score) 2. Symptoms-CDAI,RI 3. Inflammation-CRP, FCP, leucocytes	1. Moderately active IBD are capable of performing symptom-free regular endurance exercise
Cramer et al. (2017) [51]	RCT	UC Mean age = 45.5 ± 11.9	Total = 77 Intervention = 39 Control (self-written advice) = 38	Remission	Yoga - Once weekly supervised for 90 min	12 weeks	1. QoL (IBDQ) 2. Disease activity-CAI 3. Safety	1. Yoga group had significantly higher QoL 2. Disease activity was lower in the yoga group
Elsenbruch et al. (2005) [52]	RCT	UC Mean age = 40.0 ± 2.6	Total = 45 30 total IBD Intervention = 15 Control = 15 Health control = 10	Remission	Moderate exercise - Structured 60-h training program - stress management training, moderate exercise, Mediterranean diet, behavioural techniques and self-care strategies	10 weeks	1. QoL 2. Perceived stress 3. Disease activity 4. Distribution of circulating lymphocytes/lymphocyte subsets as well as the beta-adrenergic modulation of TNF-alpha production in vitro 5. Urine catecholamines and plasma cortisol, prolactin and growth hormone	1. Improvement in IBDQ-specifically in bowel symptoms 2. No difference in hormones was observed 3. No difference in perceived stress was noted 4. No significant group differences in inflammatory response
Robinson et al. (1998) [43]	RCT	CD Mean age = 45.7 ± 12.2	Total = 117 Intervention = 60 Control = 57	Remission	Main section- 12 core floor-based exercises Minimum X10 sessions/month	52 weeks	1. Effect on bone mineral density	1. Non-significant gains in BMD occurred at the hip and spine in the exercise group compared with controls 2. Compared with controls, gain in BMD at the greater trochanter was statistically significant 3. Poor compliance with the intervention
Loudon et al. (1999) [44]	Pilot prospective observational study	CD Mean age = 38.3 ± 7.5	Total = 12 (all intervention)	Remission/mildly active	Supervised walking programme X2/week	12 weeks	1. IBD stress index 2. IBD QoL Score 3. Disease activity-HBI 4. Physical fitness (the Canadian Aerobic Fitness Test, VO2 Max) 5. BMI	1. Improvement in BMI was seen 2. No patients flared
Jones et al. (2020) [45]	RCT	CD Mean age = 49.3 ± 13	Total = 47 Intervention = 23 Control = 24	‘Stable’	Combination of impact and high-effort resistance exercises X3 60-min exercise sessions on non-consecutive days 12/78 supervised	26 weeks	1. Bone mineral density 2. Muscle function 3. QoL (IBDQ) 4. Fatigue (IBD-F-scale)	1. BMD results superior with exercise and superior muscle function values
Tew et al. (2019) [46]	Pilot RCT	CD Mean age = 36.9 ± 11.2	Total = 36 HIIT = 13 MICT = 12 Control = 11	Remission/mildly active	High-intensity interval training (HIIT) and moderate-intensity continuous training (MICT)	12 weeks	1.Cardiorespiratory fitness 2. Disease activity 3. Fatigue 4. QoL 5. Adverse events 6. Intervention acceptability	1. HIIT did not exacerbate disease 2. Acceptable compliance rates 3. Few adverse events noted
Seeger et al. (2020) [47]	Pilot RCT	CD Mean age: N/a Endurance = 39.6 ± 12.0 Muscle = 42 ± 13.1 Control = 43.7 ± 12.0	Total = 45 Control = 13 Endurance training = 17 Muscle training = 15	Remission or mildly active	Bodyweight muscle training 30–40 min Endurance-walking, running or cycling 30 min X3/week, unsupervised	12 weeks	1. Dropout rate 2. Disease activity 3. Inflammatory parameters incl. FCP 4. Anthropometric data 5. QoL-(SIBDQ) 6. Physical activity and strength	1. Both exercises were safe 2. Beneficial for muscle strength and well-being 3. Higher dropout rate in the endurance group
de Souza Tajiri et al. (2014) [56]	Pilot observational study	CD and UC Average age = N/a	Total screened = 148 Total enrolled = 19 CD = 10 UC = 19	Not specified	Quadriceps training by leg extension on a weight machine X2/week, 20 min, personal trainer	8 weeks	1.Thigh circumference 2. BMI 3. Quadriceps strength parameters 4. QoL	1. Improvements in quadriceps strength 2. No change in thigh circumference 3. No exacerbations of disease 4. Improvements in intestinal component of IBDQ
van Erp et al. (2021) [57]	Pilot observational study	CD, UC or IBD-U Mean age = 45 ± 2.6	Total = 25 CD = 21 UC = 3 IBDU = 1	Remission	Aerobic and progressive resistance training at personalised intensity based on a cardiopulmonary exercise test (CPET) 1 h X3/week 30 min aerobic training (exercise bicycle, cross-trainer, or treadmill at 65–80% of the patients’ maximum heartrate) and 30 min progressive-resistance training (eight different training machines)	12 weeks	1. Fatigue 2. QoL 3. Cardiorespiratory fitness	1. Improvement in fatigue and QoL 2. Improvement in cardiorespiratory fitness
Gerbarg et al. (2015) [58]	RCT	IBD Mean age = 54 ± 15.7	Total = 29 Intervention = 16 Control = 13 UC = 9 CD = 18 IBDU = 1 Lymphocytic colitis = 1	Not- specified but median FCP values were raised at baseline	Intervention = Breath-body-mind workshop Qigong movements 9 h Then encouraged to do 20 min/day Control = education seminar	26 weeks	1. Anxiety/Depression 2. IBDQ 3. Perceived Disability Scale 4. Perceived Stress Questionnaire 5. Brief Illness Perception Questionnaire 5. Inflammation-FCP & CRP	1. Improvements in psychological and physical symptoms 2. Improvement in QoL 3. Improvement in CRP
D’incá et al. (1999) [48]	Case–control	Ileal CD Age = N/A	Total = 12 CD = 6 Healthy aged-matched controls = 6	Remission	1-h moderate physical exercise Once-off	1 h	1. Orocaecal transit time (breath test to lactulose), 2. Intestinal permeability 3. Polymorphonuclear leucocytes function 4. Lipoperoxidation 5. antioxidant trace elements	1. Moderate aerobic exercise has no significant effect on the gastrointestinal parameters examined
Mc Nelly et al. (2016) [59]	RCT	UC + CD Placebo/no exercise Median age = 31 (27, 51) Placebo/exercise Median age = 35 (28, 43) Omega 3/no exercise Median age = 45 (36, 51) Omega 3/exercise Median age = 31 (29, 55)	Total = 52 Exercise and active supplement (*n* = 11) Exercise and placebo supplement (*n* = 15) No exercise and active supplement (*n* = 14) No exercise and placebo supplement (*n* = 12)	Remission	Increase in physical activity levels of at least 30% OR initiation of walking, swimming and simple gym routines	12 weeks	1. Fatigue 2. QoL 3. BMI 4. Disease activity- CRP and clinical activity tools	1. Fatigue (IBD-F) score was reduced i.e. improved with exercise advice 2. No difference in adverse gut effects with exercise
Lamers et al. (2021) [60]	Observational study	CD and UC Mean age = 54 ± 12	IBD walkers = 18 Non-IBD walkers = 19 IBD non-walkers = 19 Total IBD = 37	Patients on biologics excluded	4 day walking march Between 30–50 km	4 days	1. Cytokine levels: IL-6, IL-8, IL-10, IL-1b, TNF-a 2. FCP 3. Validated clinical activity scores (HBI, SCCAI)	1. No differences in cytokine concentrations were found between IBD walkers and non-IBD walkers 2. No difference observed in FCP between groups 3. Clinical disease activity worsened during the exercise event in participants with CD
Gupta et al. (2005) [49]	Observational study	CD Age range (entire cohort) = 19–76 years Other dx: Asthma, HTN, Diabetes, cervical spondyloarthritis, CAD, Obesity, psychiatric problems, thyroid disease	Total = 175 GI disease including CD = 18	N/A	Yoga- total programme 3-4 h/day	8 days	1. Anxiety scores	1. There were no significant changes in anxiety scores in patients with gastrointestinal disorders
Sharma et al. (2015) [61]	RCT	UC + CD Age range = 16–60	Total IBD = 100 UC = 60 CD = 40	Remission	Intervention- 1 h Yoga daily plus standard of care Control- standard of care (Azathioprine, mesalamines, multivitamins, and Ca supplements)	8 weeks	1. Cardiovascular autonomic variability-heart rate variability 2. Parasympathetic reactivity 3. Sympathetic reactivity 4. Serum eosinophilic cationic protein 5. sIL-2R 6. Anxiety-Spielberger's State Trait Anxiety Inventory scores 7. Clinical symptoms-symptom diary	1. Fewer UC patients reported arthralgia in the yoga group 2. Intestinal colic pain in the control group was higher 3. State and trait anxiety levels were significantly reduced in UC, not CD
Langhorst et al. (2007) [53]	RCT	UC Mean age = N/A Intervention = 41.2 ± 9.9 Control = 47.3 ± 13.1	Total analysed = 56 Intervention = 30 Standard of care = 26	Remission or mild disease activity	Structured 60-h training program: Regular exercise and increased daily activity were strongly recommended, each program day included 30 min of light exercise	10 weeks	1. QoL (IBDQ) 2. Psychological distress 3. Clinical disease activity	1. A reduction in anxiety and improvement in physical function seen after 12 weeks 2. Good compliance was observed with the program 3. No significant effects on IBDQ or clinical disease parameters were observed
Spijkerman et al. (2021) [62]	Observational	UC + CD Mean age = 54 ± 12	Total = 38 IBD = 18 Non-IBD = 18	Patients on biologic therapy were excluded	Walking 30–50 km a day	3 days	1. Cellular responsiveness to fMLF in granulocytes and monocytes 2. Heart rate monitoring	1. The exercise was associated with relative refractoriness of both neutrophils and monocytes in IBD patients
Hassid et al. (2016) [63]	Pilot prospective cohort study *Conference paper	UC + CD Mean age = N/a	Total = 10 CD = 7 UC = 3	Not reported	High-intensity events -Marathon, ½ marathons, long cycles, triathlon	Once-off events	1. Clinical disease activity 2. Inflammation-FCP	1. 2 CD patients experienced an increase in symptoms (raised HBI) 2. No change in FCP pre and post events at 24 h and 1 week
Fagan et al. (2019) [64]	Prospective cohort study *Conference paper	CD + UC Mean age = 45.5 ± 16.1	Total enrolled = 82 Total analysed = 58	Remission, mild-moderate	Exercise for at least 10 min X5 days/week	16 weeks	1. PA Levels-short IPAQ 2. Disease activity-HBI/SCCAI 3. Assess compliance with exercise programme 4. Fatigue-Multidimensional Fatigue Inventory 5. Depression, anxiety QoL-IBDQ	1. IBDQ scores improved 2. High level of compliance was achieved (83%) 3. Fatigue, depression and anxiety improved 4. Mean MET-min/week increased
Benazzato et al. (2006) [50]	Prospective cohort study *Conference paper	CD Age = N/A	Total CD patients = 10 7-Ileal 3-Ileocolonic Age-matched healthy controls = 6	Remission	A treadmill test at 85% of maximal pulse rate for 55 min, at 90% of maximal pulse rate for the last 5 min	Once-off exercise Followed × 8 months for clinical relapse	1. Gastrointestinal permeability 2. Inflammation-cytokines, oxidative status	1. Basal intestinal permeability to lactulose/mannitol was higher in patients than controls and decreased significantly after exercise in patients 2. IL-6 did not change after exercise in CD patients 3. No disease relapses were observed during follow-up

*UC* ulcerative colitis, *CD* Crohn’s disease, *PA* physical activity, *QoL* quality of life, *IL* interleukin, *IBDQ* inflammatory bowel disease questionnaire, *IPAQ* international physical activity questionnaire, *HBI* Harvey Bradshaw index, *SCCAI* simple clinical colitis activity index, *MET* metabolic equivalent of task, *FCP* faecal calprotectin, *fMLF* N-Formylmethionyl-leucyl-phenylalanine, *TNF* tumour necrosis factor, *BMI* body mass index, *CRP* C-reactive protein, *DEXA* dual energy X-ray absorptiometry

### Study characteristics

**Fig. 2:**
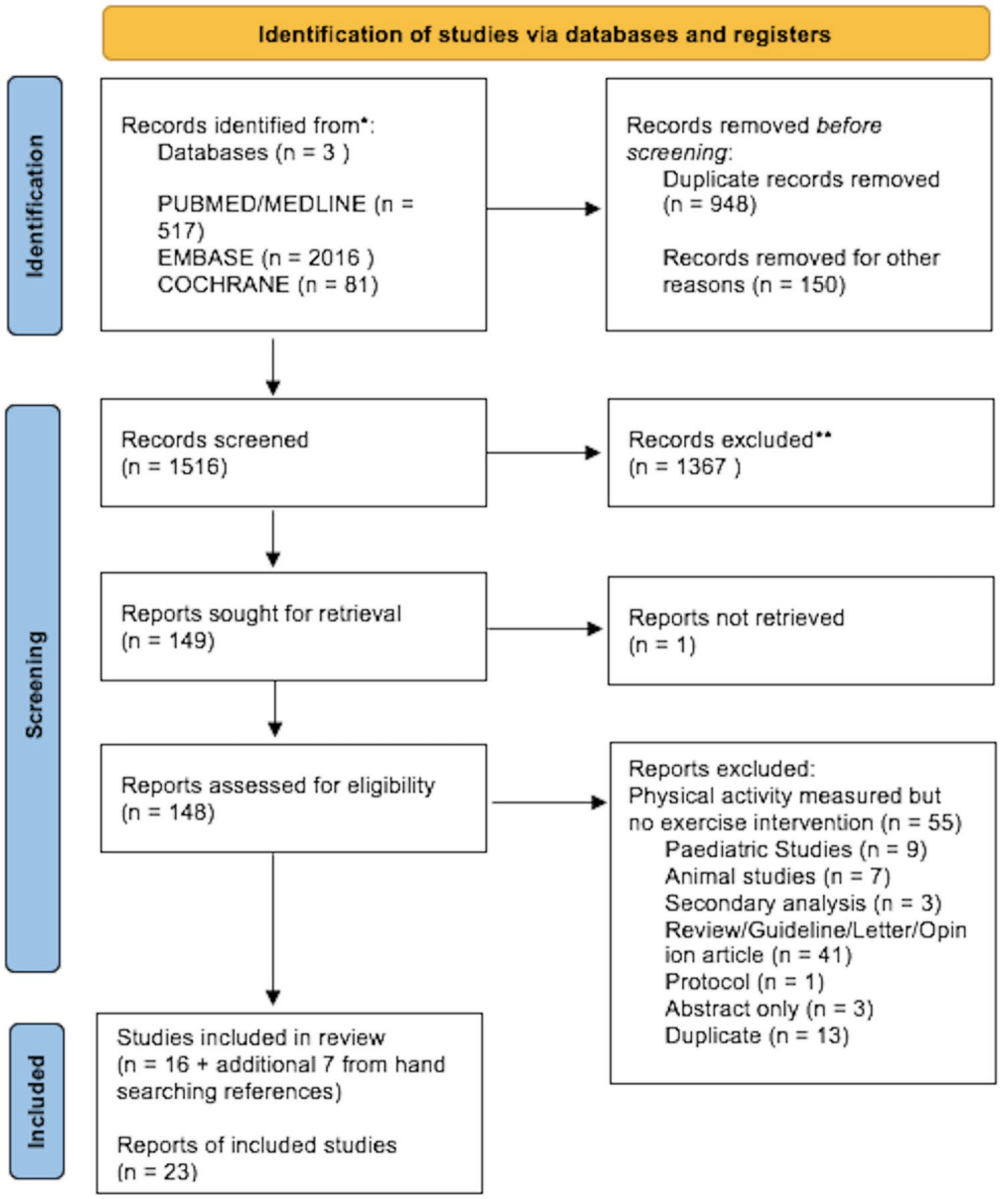
Flow diagram of included studies

In total, 1516 records were screened for eligibility, and 148 records were reviewed for eligibility (full text), of which 16 were included and a further 7 studies were identified from hand searching references (Fig. 2). Of the 23 studies, 9 studies examined the effects of structured exercise in CD [42–50], 3 studies examined the effects in UC [51–53], and 11 studies examined the effects in unspecified patients with IBD [54–64]. Patients were in clinical remission or had mildly active disease in 15 studies [42–48, 50–54, 57, 59, 61]; two studies included patients with moderate disease [55, 64]; in 4 studies, the disease activity was unspecified [49, 56, 58, 63]; and 2 studies excluded patients on biologic therapies [60, 62]. Of the studies included, 11 were randomised controlled trials [42, 43, 45, 51, 52, 54, 55], 2 were pilot RCTs [46, 47], and the remaining 10 studies were observational (prospective cohort or case-control) [44, 48, 49, 56, 57, 60, 62, 63].

### Exercise programmes and overview of outcomes measured

The types of structured exercise programmes ranged from combined aerobic and resistance training (*n* = 7) to walking (*n* = 6), running (*n* = 1), yoga (*n* = 3), high and moderate intensity interval training (*n* = 6), Qigong (*n* = 1) and swimming (*n* = 1). The outcomes of the studies varied from assessments of quality of life, disease activity, inflammatory response, body composition, physical fitness, mental health outcomes, feasibility measurements and fatigue. Programmes that included combined aerobic and resistance training demonstrated an increase in physical fitness and high compliance rates and were deemed safe with no significant deterioration in disease activity [45, 54, 57]. Both high and moderate-intensity interval training were deemed to have acceptable safety profiles [46, 52, 55] in addition to walking interventions which also led to an improvement in patient quality of life [42, 44, 64]. Yoga and Qigong [49, 51, 58, 61] were largely beneficial for improving psychological symptoms and quality of life without demonstrating any specific benefit in terms of disease control. Higher drop-out rates were seen in the study by Seeger et al. in the endurance exercise group due to “loss of motivation’ and “bad weather” [47].

### Duration of the exercise programmes

The duration of exercise programmes varied from a once-off treadmill test to a year-long structured programme [43, 50]. The majority of programmes were from 8 to 12 weeks in duration [42, 44, 46, 47, 51–57, 59, 61, 64]. The majority of studies of 12-week duration or more found an improvement in quality of life and fatigue scores were measured [42, 51, 57–59, 64]. A number of studies reported once-off exercise events including walking marches, marathons, triathlons and a treadmill test [48, 50, 60, 62, 63] with outcomes focusing on the inflammatory response to the exercise which will be discussed later (Table 4). The longest study of 52 weeks reported a difference in compliance of 7% less at 1 year for the exercise arm versus controls; however, the compliance rate remained high at 88% for the exercise group [43].

### Body composition analysis in exercise studies in IBD patients

Of the studies included in this systematic review, 4 studies examined the effect of structured exercise on body composition (Table 3). The use of dual energy X-ray absorptiometry (DEXA) was the most commonly used imaging modality for the determination of body composition measurements [43, 45, 54]. The 3 studies included which used DEXA were all randomised controlled trials in design. The focus of body composition measurements differed between studies with Cronin and colleagues focusing on total fat mass, truncal fat mass, body fat percentage and total lean tissue mass, whereas publications by Robinson et al. and Jones et al. focused on bone mineral density measured by DEXA [43, 45, 54]. Cronin and colleagues carried out an 8-week aerobic and resistance exercise programme of moderate-intensity in patients with IBD with quiescent or mild disease. The primary outcome of the study was to determine a change in body composition of patients participating in the exercise programme. Results of the study found that favourable changes in body composition were observed in the exercise group with a median decrease in total body fat percentage of 2.1% versus a mean gain of 0.1% body fat in the control group (*p* = 0.022). In addition to the changes in body fat percentage, an increase in total lean tissue mass of 1.59 kg was observed in the exercise group compared to 1.38 kg among controls (*p* = 0.003) [54].

**Table 3:** Results of body composition analysis

**Study type**		**Author and date**	**Study design**	**Purpose**	**Sample**	**Type of exercise**	**Duration of study**	**Imaging modality/protocol**	**Finding**	**Risk of bias**
**Body composition**	1	Cronin et al. (2019) [54]	RCT	Total fat mass, truncal fat mass, total lean tissue mass and percentage body fat were recorded	20 patients with IBD in remission	Aerobic and resistance Moderate intensity	8 weeks	DEXA Body composition using a three-compartment model (fat mass, bone mass, lean tissue)	Change in body composition in the exercise group - 2.1% reduction in total % body fat - Median 1.59 kg increase in total lean tissue mass	Some concerns
	2	Robinson et al. (1998) [43]	RCT	Effects of exercise on bone mineral density	117 CD	Core floor exercises	52 weeks	BMD at the hip and lumbar spine measured by DEXA at baseline and 12 months	BMD increased at all measured sites in the training group, however this did not reach clinical significance Compared with controls, gain in BMD at the greater trochanter was statistically significant	Some concerns
	3	Jones et al. (2020) [45]	RCT	Effects on bone mineral density	47 ‘Stable’ CD	Combination of impact and high-effort resistance exercises	26 weeks	BMD measured by DEXA at the hip & lumbar spine at baseline and 6 months	BMD values for the exercise group were superior to those for the control group with statistical significance at lumbar spine (*p* < 0.001) but not at the hip	Some concerns
	4	De Souza Tajiri et al. (2014) [56]	Pilot observational study	Thigh circumference	19 IBD patients	Quadriceps training by leg extension on a weight machine	8 weeks	Measured manually in mm at baseline and 8 weeks	No change was observed in thigh circumference	Moderate

*RCT* randomized controlled trial, *IBD* inflammatory bowel disease, *BMD* bone mineral density, *CD* Crohn’s disease, *UC* ulcerative colitis, *DEXA* dual energy X-ray absorptiometry, *mm* millimetres

Both Robinson et al. and Jones et al. aimed to examine the effect of structured exercise on bone mineral density (BMD) using DEXA. Robinson and colleagues aimed to determine the effect of low impact exercise on BMD over 12 months by dynamically loading the axial skeleton and the hip and lumbar spine with at-home, low impact exercises (predominantly core-based exercises). BMD was measured at the hip and lumbar spine at baseline and at 12 months. Although a non-significant increase was observed in BMD at the hip and spine in the exercise group versus controls (*p* = 0.05), the gain in BMD at the greater trochanter was significantly superior to that of controls (difference in means, 4.67; 95% confidence interval: 0.86–8.48; *p* = 0.02). The greater number of exercises carried out, the greater the gain in BMD (femoral neck: *r*^2^ = 0.28; 95% confidence interval: 0.10–0.45; *p* = 0.04) [43]. In comparison, Jones et al. used combined impact and resistance training to illicit changes in BMD in patients with stable CD. Exercises included were of a higher impact compared to that utilised by Robinson et al. A 6-month period was assigned for follow-up, and at this timepoint, BMD values were observed to be superior in the exercise group at the lumbar spine (adjusted mean difference 0.036 g/cm^2^, 95% CI 0.024–0.048; *p* < 0.001), but not at femoral neck (0.018 g/cm^2^, 0.001–0.035; *p* = 0.059) or greater trochanter (0.013 g/cm^2^, − 0.019 to 0.045; *p* = 0.415) after correcting for multiple outcomes.

DeSouza et al. adapted a different approach to the inclusion of body composition measurements by measuring thigh circumference manually in response to progressive quadriceps resistance training in female patients with IBD. 19 patients were included (10 = CD, 9 = UC) who carried out an 8-week machine-based quadricep training programme. An increase in thigh strength greater than 40% was observed; however, thigh circumference did not increase from baseline to the time of programme completion at 8 weeks. The authors suggest that the increase in thigh strength was likely relative to neural adaptation rather than muscle hypertrophy itself [56].

### Inflammatory response to exercise in IBD

In total, we include 14 studies which objectively measure the inflammatory response to structured exercise programmes (Table 4). Seven studies examined the response of C-reactive protein (CRP) to exercise [46, 47, 51, 54, 55, 58, 59], and only one study, which also included patients with lymphocytic colitis, demonstrated a statistically significant improvement in CRP after Qigong from baseline, with the CRP remaining elevated post the exercise intervention (730.0 versus 836.0 ng/mL, respectively, *p* = 0.01) [58]. We note that faecal calprotectin (FCP) response was not observed in the same study [58]. Similarly, in the vast majority of other studies examining the effects of exercise on FCP, no significant improvement was observed in response to the studied intervention [46, 47, 51, 55, 63]. On the contrary, in the study by Tew et al., the FCP was elevated in two patients; however, one of these patients already had an elevated FCP in the few months prior to the exercise intervention [46].

**Table 4:** Results of inflammatory response to exercise in IBD patients

**Study Type**		**Author and date**	**Study design**	**Type of exercise**	**Sample/disease activity**	**Inflammatory markers measured**	**Timing of measurements**	**Findings**	**Risk of bias**
**Inflammation**	1	Cronin et al. (2019) [54]	RCT	Aerobic and resistance Moderate intensity	20 patients with IBD in remission	CRP Cytokines IL-6, IL-8 and IL-10, TNF-α	Baseline and 8 weeks	No significant change in cytokines or CRP pre and post exercise intervention	Some concerns
	2	Klare et al. (2015) [55]	RCT	Moderate intensity running	30 mild-mod IBD	CRP Leucocytes FCP	Baseline and 10 weeks	1. Statistically insignificant change in FCP 2. No significant change in CRP 3. Significant change in leucocytes within the exercise group *p* = 0.016	Some concerns
	3	Cramer et al. (2017) [51]	RCT	Yoga	77 UC in remission	CRP, ESR, Leucocytes FCP Faecal PMN elastase Faecal lactoferrin	Weeks 1, 12 and 24	No significant change was seen between groups	Some concerns
	4	Elsenbruch et al. (2005) [52]	RCT	‘Moderate’ exercise	30 UC in remission	Leucocytes Lymphocyte subsets helper T cells (CD3 + CD4 + lymphocytes), cytotoxic/suppressor T cells (CD3 + CD8 + lymphocytes), natural killer (NK) cells (CD3–CD16 + CD56 + lymphocytes), B-cells (CD3–CD20 + lymphocytes) and monocytes (CD14 + leukocytes) TNF-α response to in-vitro B-adrenergic agonist	Baseline and 10 weeks	1. No significant effects of therapy on either the basal levels of TNF- or on the suppressive effect of isoproterenol on TNF-production were observed 2. No significant effects of therapy were found in absolute numbers of circulating lymphocyte subsets	Some concerns
	5	Tew et al. (2019) [46]	Pilot RCT	HIIT/MICT	39 mildly active CD	FCP T lymphocyte subsets TH1, TH2, TH17 Cytokines IL-6, IL-10, TNF-α CRP	Baseline and 12 weeks	1. 2 patients flared with rise in FCP > 400 ug/g (one HIIT- had a FCP > 400 in previous 6 months, one MICT) 2. Formal analysis of T Lymphocytes and Cytokines has not been carried out to date	Some concerns
	6	Seeger et al. (2020) [47]	RCT	Bodyweight muscle training Endurance-walking, running or cycling	45 Remission/mildly active CD	FCP CRP Leucocytes	Baseline and 12 weeks	No statistically significant difference in inflammatory markers	Some Concerns
	7	Gerbarg et al. (2015) [58]	RCT	Qigong	29 IBD/Lymphocytic colitis	CRP FCP	Baseline, weeks 6 and 26	1. Significant improvement in CRP in the intervention group compared with baseline (730.0 versus 836.0 ng/mL, respectively, *p* = 0.01) 2. No significant change in FCP	Some concerns
	8	D’incá et al. (1999) [48]	Case–control	Moderate physical exercise	6 CD in remission	PMN leukocyte function	Same day before and after exercise	Neutrophils, primed pre exercise in Crohn's disease patients showed an increased post exercise chemiluminescence similar to controls	Serious
	9	Mc Nelly et al. (2016) [59]	RCT	Increase PA by 30% or initiate exercise programme	52 IBD in remission	CRP	Baseline and 12 weeks	No significant change in CRP	Some concerns
	10	Lamers et al. (2021) [60]	Observational/case–control	4-day walking march Continuous moderate intensity activity	39 IBD not on biologics	Cytokines (IL-6, IL-8, IL-10, IL-1β and TNF-α) FCP	1–2 days prior the event 30 min after completion exercise days 1–4	1. No differences in cytokine concentrations were found between IBD walkers and non-IBD walkers BUT temporary significant increase in IL-6 (*p* < 0.001) and IL-10 (*p* = 0.006) from baseline to post exercise day 1 2. No difference in FCP between groups	Moderate
	11	Sharma et al. (2015) [61]	RCT	Yoga	100 IBD in remission	Serum eosinophilic cationic proteins Serum IL-2 soluble receptors	Baseline, weeks 4 and 8	No significant changes in immunological markers	Some Concerns
	12	Spijkerman et al. (2021) [62]	Observational	Walking × 3 days 30–50 km/day	18 IBD pts not on biologics 10 UC 8 CD	Responsiveness of neutrophils, eosinophils and monocytes to fMLF	Baseline and after 3 days of repeated exercise	At day 3 ↑ responsiveness of neutrophil and monocytes was found in all walkers (IBD and non-IBD), but was significantly less increased in IBD patients.	Moderate
	13	Hassid et al. (2016) [63]	Observational	High-intensity exercise	10 IBD patients 7 CD 3 UC	FCP	24 h pre-event, 24 h post-event, 1 week post-event	FCP did not significantly elevate post events.	Serious
	14	Benazzato et al. (2006) [50]	Observational	Moderate intensity on treadmill	10 CD patients in remission	IL-6 Plasmatic endotoxin Total plasmatic antioxidant status (BAP test) Total oxidative status (dROMs test)	Data at rest and after exercise	No change in IL-6 in CD patients, plasma endotoxin or total oxidative stress post exercise.	Moderate

*IBD* inflammatory bowel disease, *UC* ulcerative colitis, *CD* Crohn’s disease, *CRP* C-reactive protein, *FCP* faecal calprotectin, *BAP test* biological antioxidant potential, *dROMs test* reactive oxygen metabolites, *IL* interleukin, *fMLF* N-Formylmethionyl-leucyl-phenylalanine, *TNF* tumour necrosis factor, *PMN* polymorphonuclear leucocyte, *ESR* erythrocyte sedimentation rate

Four studies examined the effects of exercise (prolonged walking, running and combined aerobic-resistance training) on pro-inflammatory cytokine levels, of which three studies carried out a formal analysis including of IL-6, IL-8, IL-10, IL-1β and TNF-α pre and post exercise [50, 54, 60]. Lamers et al. found that there was no difference in IL-6, IL-8, IL-10, IL1b or TNF-a in a mixed linear model but that a transient increase in both IL-6 and IL-10 was observed between IBD-walkers and non-IBD non-walkers at baseline and post day 1 exercise during a 4-day walking march. The authors conclude that exercise of moderate intensity caused a rise in the pro-inflammatory cytokine expression rather than IBD disease activity itself [60].

More novel markers of inflammatory activity measured in other studies included T-lymphocyte subsets, serum eosinophilic cationic proteins, faecal polymorphonuclear neutrophil elastase, faecal lactoferrin and markers of immunometabolism (plasma endotoxin, oxidative stress) [46, 50–52, 61, 62]. Studies by Elsenbruch et al. and Tew et al. included the effects of their exercise programmes on circulating T lymphocytes [46, 52]. Elsenbruch et al. examined an array of lymphocyte subsets in UC patients in remission in response to a moderate 10-week exercise programme and found no substantial effect of the exercise therapy on helper T cells (CD3 + CD4 + lymphocytes), cytotoxic/suppressor T cells (CD3 + CD8 + lymphocytes), natural killer (NK) cells (CD3– CD16 + CD56 + lymphocytes), B-cells (CD3–CD20 + lymphocytes) or monocytes (CD14 + leukocytes) [52]. To date, formal analysis on circulating T lymphocytes has not been completed.

Cramer and colleagues did not observe a change in these faecal markers during a 24 week yoga programme in UC patients who were in clinical remission [51].

Sharma et al. carried out an observational study examining the effects of an 8-week yoga programme on IBD patients which included the observation of the change in both serum eosinophilic cationic proteins and soluble interleukin-2 receptors (induce T-lymphocyte cytotoxicity and stimulates natural killer cell activity [65]) in response to the programme. Neither markers changed significantly as a result of the programme which included 100 IBD patients in clinical remission.

Finally, Benazatto et al. observed the effect of a once-off ‘moderate intensity’ treadmill exercise on antioxidant status in patients in remission with Crohn’s disease. Plasmatic endotoxin, total plasmatic antioxidant status and total oxidative status were assessed by enzyme-linked immunosorbent assay (ELISA), chromogenic limulus amoebocyte lysate (LAL) and spectrophotometry, respectively, at rest and after exercise. Whilst a higher intestinal permeability to lactulose/mannitol was observed in patients with CD versus healthy controls, no significant difference was recorded in plasma endotoxin levels between the two groups [50].

The type and duration of exercise differs significantly between the studies included. The type of exercise varies from walking to yoga, running, Qigong and interval training. The inclusion of IBD disease-types also differs between studies, with some including all IBD patients and others limiting the studies to either UC or CD. Furthermore, the inflammatory markers measured differ significantly between studies. For all studies excluding 1, patients were in remission or had mildly active disease which may have impacted on results of inflammatory markers in the absence of clinically active disease states [55]. Hence, significant heterogeneity exists between studies, and therefore, it was not possible to carry out a meta-analysis.

### Risk of bias and quality assessment

#### Risk of bias

**Fig. 3:**
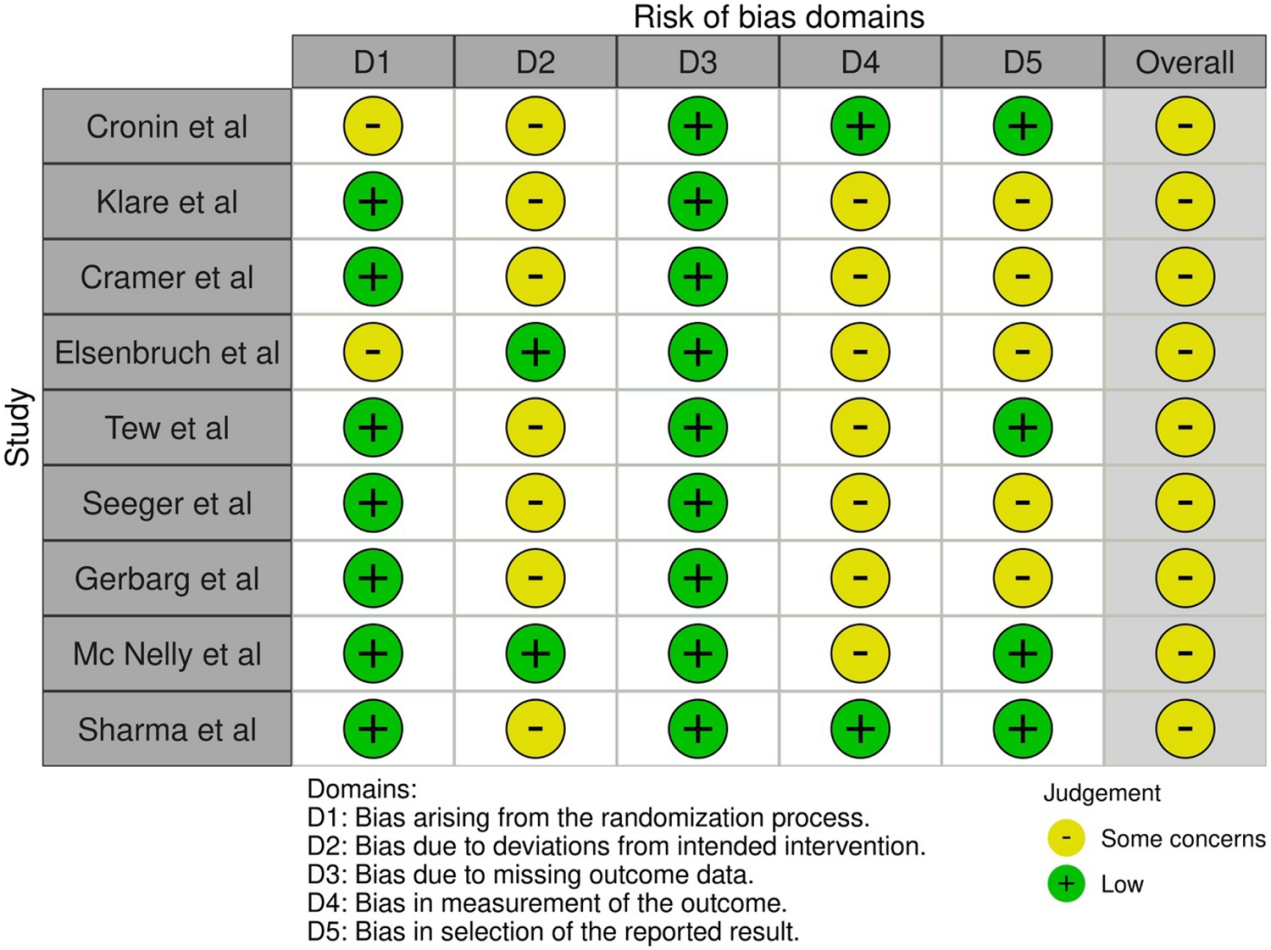
Risk of bias graph for inflammatory response to exercise in RCTs

**Fig. 4:**
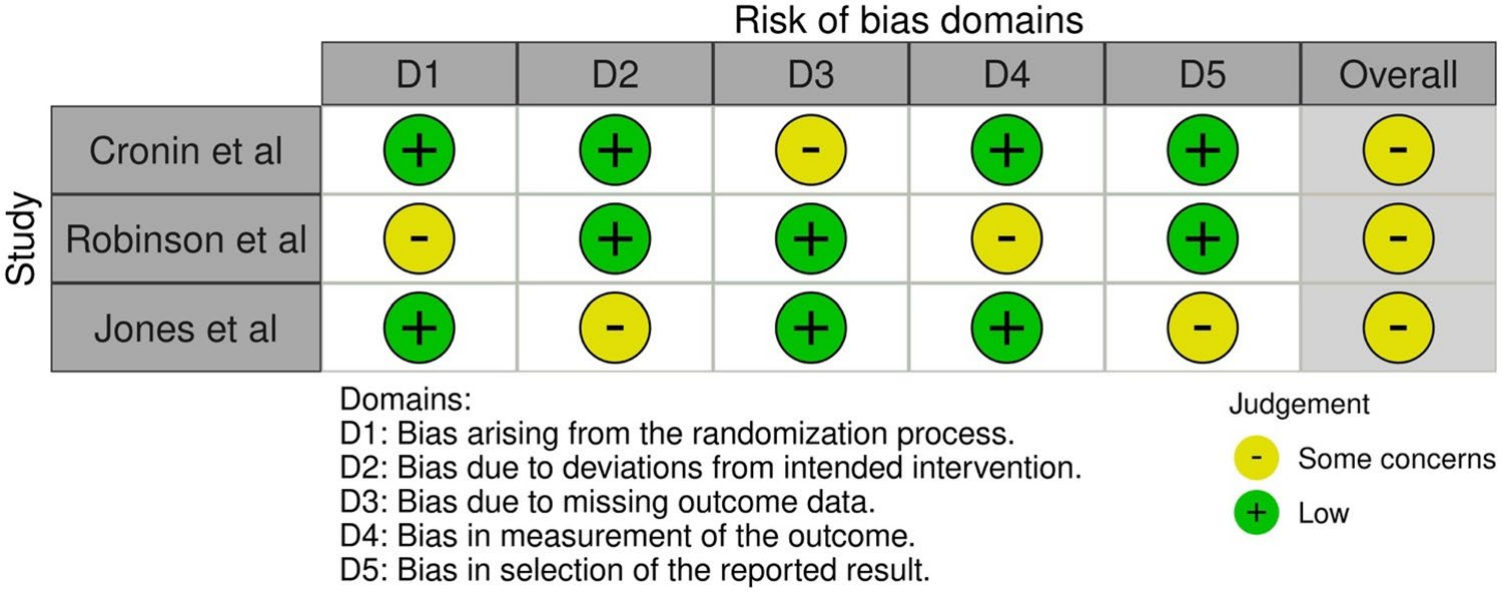
Risk of bias graph for body composition in RCTs

The risk of bias was assessed using the ROB 2.0 tool for 11 RCTs which were all deemed to have some concerns for bias for both inflammatory and body composition outcomes [43, 45–47, 51, 52, 54, 55, 58, 59, 61] (Figs. 3 and 4). The absence of pre-published study protocols for a number of studies meant there may be bias due to deviations in the intended interventions. Not at all studies followed the intention-to-treat principle. The lack of blinding of investigators in some studies may also have led to an increase in bias.

The ROBINS-I tool was used to evaluate the risk of bias in the included observational studies examining body composition and inflammatory response to measured exercise interventions. Six observational studies were included none of which were deemed to be of low risk of bias [48, 50, 56, 60, 62, 63]. Common areas for concern for bias were due to confounding and selection of participants with two studies being deemed as serious risk (Supplementary Figs. 1 and 2).

#### Quality of evidence

The GRADE (Grades of Research, Assessment, Development and Evaluation) online assessment tool was used to analyse the certainty of evidence for both outcomes (both body composition changes in response to exercise and inflammatory response to exercise). Results of body composition assessments in response to exercise using DEXA were reported in 3 separate randomised controlled trials with a risk of bias identified due to the lack of blinding given the nature of the intervention, single operator status and a significant difference in exercise type between studies. However, the overall quality of evidence was deemed to be moderate. Only one study by Cronin et al. used DEXA imaging to examine the effects of exercise on body and lean muscle composition [54]. The quality of evidence was also reviewed for the inflammatory response to the exercise interventions for 9 RCTs and 5 observational studies. Significant differences in patient selection and type of exercise were identified between the RCTs. Allocation concealment was often unclear, and there were significant differences in the type of inflammatory markers measured between studies; therefore, the level of certainty of evidence was deemed low (Table 5).

**Table 5:** GRADE assessment

**Outcomes**	**№ of participants (studies)** **Follow-up**	**Certainty of the evidence** **(GRADE)**
DEXA assessed body composition (body composition) assessed with DEXA follow-up: range 2 months to 12 months	170 (3 RCTs)	⨁⨁⨁◯ Moderate^a,b,c,d^
Inflammatory response RCTs assessed with inflammatory marker follow-up: range 8 weeks to 26 weeks	339 (9 RCTs)	⨁⨁◯◯ Low^b,c,e^
Muscle thickness measurement (MT) assessed with manual thickness measurement follow-up: mean 8 weeks	19 (1 RCT)	⨁⨁⨁◯ Moderate^b,d,f^
Inflammatory response and observational studies assessed with inflammatory markers	130 (5 observational studies)	⨁⨁◯◯ Low^b,c,f^

^a^The types of exercise performed to assess changes in BMD differed significantly between studies. Only 1 study (Cronin et al.) used DEXA to determine body fat and lean muscle mass composition

^b^Due to the nature of the intervention, patients and investigators could not be blinded

^c^Significant differences between the types of exercise carried and IBD patients are evident and included, respectively

^d^Concern regarding single operator and the external validity

^e^Not all data has been analysed for inflammatory outcomes

^f^A control arm is not included

## Discussion

Current peer-reviewed studies of the impact of exercise among IBD patients range from low intensity exercises such as yoga, Qigong and walking [42, 51, 58] to moderate intensity aerobic and resistance training [52, 54, 55] to high-intensity interval and impact resistance training [45, 46, 57, 63]. This systematic review was carried out to focus primarily on the inflammatory response to structured exercise in patients with IBD and body composition changes observed in response to structured exercise in this cohort of patients. Previous demonstrated benefits of exercise in patients with IBD include an improvement in quality of life, fatigue, cardiorespiratory fitness and confirmation of the safety of various exercises in mild to moderate disease [46, 47, 54, 59, 64]. A systematic review of the exercise-induced inflammatory response to IBD, published post registration of this current review, focused on 5 studies only and did not use the Cochrane tools for risk of bias [28]. A recently published systematic review and meta-analysis by Jones et al. reported an overview of physiological and psychological outcomes in adult IBD patients in response to exercise programmes of at least 4-week duration; hence, it did not include studies of shorter duration, which excluded the review of a number of studies that examine novel biomarkers of inflammation which is a strength of this current review.

Few studies included in the review examined the potential anti-inflammatory of exercise in addition to changes in body composition.

In a short follow-up period of 8 weeks, Cronin and colleagues demonstrated favourable changes in body composition in the exercise group compared with healthy controls, whereas no demonstrable change in pro-inflammatory cytokines was observed with exercise. Patients included were known to have quiescent or mild disease which may account for the lack of inflammatory response to the prescribed exercise in addition to the small sample size of patients enrolled. A reduction in body fat itself could account for an anti-inflammatory response and reduction in pro-inflammatory cytokines due to the pro-inflammatory nature of adipose tissue [30, 33, 66, 67]. We observed that the baseline BMI for active patients in this study was in the ‘overweight’ but not obese category with a mean BMI of 28 kg/m^2^. Previous studies have shown a relationship between higher BMI and raised CRP in CD [68] and the presence of visceral fat, fat wrapping and a raised visceral: subcutaneous fat ratio are associated with a more complicated disease course, higher postoperative complications and post-operative recurrence of CD [31, 69, 70]. Given the clinical importance of visceral and mesenteric fat in these patients, future research looking at the body composition changes associated with structured exercise should include these as standard body composition outcomes given that available studies did not address these parameters.

Both Robinson et al. and Jones et al. examined the effect of exercise on bone mineral density (BMD) in patients with CD. Robinson et al. observed that significant gains in BMD at the greater trochanter were statistically significant in the exercise group who carried out core exercises (*p* = 0.02), whereas the same was not observed at the hip and spine, potentially due to poor compliance with the exercise reported by the authors [43]. In comparison, Jones et al. demonstrated that combined impact and resistance training improved BMD at the lumbar spine (*p* < 0.001), but the same effect was not seen at the femoral neck or greater trochanter; however, muscle function outcomes were also superior in the exercise group versus controls (*p* < 0.001). Our results are similar to those reported in the review by Jones et al. [71].

A wide variety of inflammatory biomarkers were assessed in the current exercise studies in IBD. Given the majority of studies included patients with mild or quiescent disease, it is unsurprising that statistically significant changes in biomarkers in response to exercise were rarely observed. Only one study demonstrated a change in CRP, and it is unlikely the observed reduction in CRP was as a result of the exercise intervention [58]. The inclusion of studies of short duration or once-off events allowed for the review of a number of novel inflammatory biomarkers. Faecal polymorphonuclear neutrophil elastase and faecal lactoferrin have been shown in prior studies to correlate with the clinical activity index (CAI) score and to differentiate mucosal healing versus endoscopic inflammation in UC [72]. Eosinophil infiltration in the lamina propia has been included in the Geboes histological score for UC as eosinophils have been identified as significant contributor cells to immune infiltration in IBD [73]. Additionally, one of the hallmarks in the early diagnosis of IBD is the presence of eosinophilia-associated basal plasmacytosis [74]. Reports suggest that circulating endotoxin as a result of increased intestinal permeability is found in IBD and associated with increasing disease severity [75]. Novel biomarkers of inflammation included in studies in this review were pro-inflammatory cytokines, circulating T-lymphocyte subsets, serum eosinophilic cationic proteins, faecal polymorphonuclear neutrophil elastase, faecal lactoferrin and markers of immunometabolism [51, 52, 54]. Whilst the studies included did not demonstrate significant changes in the biomarkers investigated as a response to exercise, they are useful to include to inform biomarker panel development for future studies in the appropriate patient population. We suggest that the exclusion of patients with active disease, including those on biologic therapy, and the duration and nature of the exercises (yoga and walking) are the most likely factors leading to a lack of significant response of inflammatory biomarkers to the exercise programmes.

The duration of exercise programmes is an important factor to consider in order to demonstrate positive effects on inflammation, body composition and habit formation. We previously noted that studies on the effect of exercise on the microbiome that were of too short duration did not demonstrate significant changes in gut microbiome composition, diversity and functionality [76]. The study by Cronin et al. which was of 8-week duration, did not show any significant difference in gut microbiome composition/diversity or in pro-inflammatory markers in response to the exercise programme [54], whereas exercise studies in other populations of at least 12-week duration have demonstrated positive microbiome effects [77, 78]. Given the proven safety of a variety of exercises in patients with IBD, we suggest future studies examining the effects of exercise in IBD should be of at least 12-week duration and of moderate intensity to comply with the current World Health Organization guidelines on physical activity [18].

Limitations of the studies reviewed include significant differences in type and duration of exercise, the inclusion of predominantly patients who were in remission or mildly active disease, heterogeneity between inflammatory markers measured and a lack of blinding of participants and investigators. It is clear that participants carrying out the exercise intervention cannot be blinded to the intervention; however, this may lead to bias when participants complete patient-reported outcome measures; therefore, standardised biomarkers would be useful in addition to blinding of investigators who are interpreting the results. Small samples sizes were seen in the majority of studies and in the pilot studies included which lead to concern regarding adequate power of the studies and may explain the lack of significant changes particularly in inflammatory markers in response to the interventions. We acknowledge that the authors of the individual studies published the analysed results, despite often a lack of favourable outcomes, thus reducing the presence of publication bias.

The strengths of the review are the inclusion of novel studies examining inflammatory biomarkers in response to exercise in IBD, the use of robust reporting and search tools including the Cochrane guidelines (including the Cochrane risk of bias reporting tools for both RCTs and observational studies) and the MECIR manual and the PRISMA search guidelines; registration of the protocol a priori and our reporting was strengthened by the participation of a librarian from our institution’s library. Limitations of the study include a search using the English language only, the exclusion of studies in adolescents may have led to the exclusion of data relevant to the adult IBD population, and given the heterogeneity of the type of study, interventions and outcomes, a meta-analysis was not carried out.

## Conclusion

The quality of evidence for a significant inflammatory response to the current exercise studies in IBD is low. Adequately powered studies of adequate duration with the inclusion of patients with active disease are required to assess disease-altering changes in the microbiome which in turn may yield anti-inflammatory effects through the modulation of immuno-metabolic pathways. The development of a biomarker panel would help to standardise reporting of the inflammatory response in future studies.

This review has shown a moderate level of evidence supporting the positive body composition changes as a result of exercise in patients with IBD which supports the findings of previous reviews, although very few studies examined these changes and further adequately powered studies are required.

The majority of studies included patients with mild or quiescent IBD; hence, further studies are required to include patients with more active disease, including those on biologic or small molecule therapies, which is more reflective of real-world patients. In conclusion, the results of this review suggest limited evidence to support the anti-inflammatory effect of exercise programmes in patients with IBD based on data published to date but demonstrate the positive effect of exercise on body composition. Exercise programmes in IBD remain a promising area for future research and as an adjunctive therapy in inflammatory bowel disease management.

### Supplementary Information

Below is the link to the electronic supplementary material.

Supplementary file1 (DOCX 1005 KB)

## References

[1] Zhang YZ, Li YY (2014). Inflammatory bowel disease: pathogenesis. World J Gastroenterol.

[2] Torres J (2017). Crohn's disease. Lancet.

[3] Jairath V, Feagan BG (2020). Global burden of inflammatory bowel disease. Lancet Gastroenterol Hepatol.

[4] Gecse KB, Vermeire S (2018). Differential diagnosis of inflammatory bowel disease: imitations and complications. Lancet Gastroenterol Hepatol.

[5] Abraham C, Cho JH (2009). Inflammatory bowel disease. N Engl J Med.

[6] McDermott E (2015). Body image dissatisfaction: clinical features, and psychosocial disability in inflammatory bowel disease. Inflamm Bowel Dis.

[7] Targan SR et al (1997) A short-term study of chimeric monoclonal antibody cA2 to tumor necrosis factor alpha for Crohn's disease. Crohn's Disease cA2 Study Group. N Engl J Med 337(15):1029–35 10.1056/NEJM199710093371502 9321530

[8] Colombel JF (2010). Infliximab, azathioprine, or combination therapy for Crohn’s disease. N Engl J Med.

[9] Torres J (2020). ECCO guidelines on therapeutics in Crohn’s disease: medical treatment. J Crohns Colitis.

[10] Harbord M et al (2017) Third European evidence-based consensus on diagnosis and management of ulcerative colitis. Part 2: Current Management. J Crohn Colit 11(7):769–784 10.1093/ecco-jcc/jjx009 28513805

[11] Ford AC et al (2011) Efficacy of biological therapies in inflammatory bowel disease: systematic review and meta-analysis. Am J Gastroenterol 106(4):644–59, quiz 660 10.1038/ajg.2011.73 21407183

[12] Singh S (2020). First- and second-line pharmacotherapies for patients with moderate to severely active ulcerative colitis: an updated network meta-analysis. Clin Gastroenterol Hepatol.

[13] Stidham RW (2014). Systematic review with network meta-analysis: the efficacy of anti-TNF agents for the treatment of Crohn's disease. Aliment Pharmacol Ther.

[14] Chen C (2019). Real-world pattern of biologic use in patients with inflammatory bowel disease: treatment persistence, switching, and importance of concurrent immunosuppressive therapy. Inflamm Bowel Dis.

[15] Ben-Horin S, Kopylov U, Chowers Y (2014). Optimizing anti-TNF treatments in inflammatory bowel disease. Autoimmun Rev.

[16] Torres J (2019). European Crohn’s and colitis organisation topical review on complementary medicine and psychotherapy in inflammatory bowel disease. J Crohns Colitis.

[17] Eckert KG (2019). Structured physical activity interventions as a complementary therapy for patients with inflammatory bowel disease - a scoping review and practical implications. BMC Gastroenterol.

[18] Bull FC (2020). World Health Organization 2020 guidelines on physical activity and sedentary behaviour. Br J Sports Med.

[19] Pescatello LS, Ross A, Deborah R (2013) General principles of exercise prescription. In: Pescatello LS, Arena R, Riebe D, Paul D (eds) Guidelines for exercise testing and presciption, 9th edn. Thompson, Philadelphia, PA, pp 162–193

[20] Roig M (2009). The effects of eccentric versus concentric resistance training on muscle strength and mass in healthy adults: a systematic review with meta-analysis. Br J Sports Med.

[21] Patel H (2017). Aerobic vs anaerobic exercise training effects on the cardiovascular system. World J Cardiol.

[22] Gleeson M (2011). The anti-inflammatory effects of exercise: mechanisms and implications for the prevention and treatment of disease. Nat Rev Immunol.

[23] Petersen AM, Pedersen BK (2005) The anti-inflammatory effect of exercise. J Appl Physiol (1985) 98(4):1154–62 10.1152/japplphysiol.00164.2004 15772055

[24] Akdis M (2016). Interleukins (from IL-1 to IL-38), interferons, transforming growth factor β, and TNF-α: receptors, functions, and roles in diseases. J Allergy Clin Immunol.

[25] Papadakis KA, Targan SR (2000). Role of cytokines in the pathogenesis of inflammatory bowel disease. Annu Rev Med.

[26] Cui G (2021). Evaluation of anti-TNF therapeutic response in patients with inflammatory bowel disease: current and novel biomarkers. EBioMedicine.

[27] Pedersen BK (2000). Special feature for the Olympics: effects of exercise on the immune system: exercise and cytokines. Immunol Cell Biol.

[28] Baker KA (2022). The exercise-induced inflammatory response in inflammatory bowel disease: a systematic review and meta-analysis. PLoS ONE.

[29] Bryant RV (2019). Visceral adipose tissue is associated with stricturing Crohn’s disease behavior, fecal calprotectin, and quality of life. Inflamm Bowel Dis.

[30] Rowan CR, McManus J, Boland K, O'Toole A (2021) Visceral adiposity and inflammatory bowel disease. Int J Colorectal Dis 36(11):2305–2319. 10.1007/s00384-021-03968-w. Epub 2021 Jun 9. PMID: 34104989 10.1007/s00384-021-03968-w 34104989

[31] Ding Z (2016). Association between high visceral fat area and postoperative complications in patients with Crohn’s disease following primary surgery. Colorectal Dis.

[32] Ding NS (2017). The body composition profile is associated with response to anti-TNF therapy in Crohn’s disease and may offer an alternative dosing paradigm. Aliment Pharmacol Ther.

[33] Fink C (2012). Adipose tissue and inflammatory bowel disease pathogenesis. Inflamm Bowel Dis.

[34] Harper JW, Zisman TL (2016). Interaction of obesity and inflammatory bowel disease. World J Gastroenterol.

[35] Shuster A (2012). The clinical importance of visceral adiposity: a critical review of methods for visceral adipose tissue analysis. Br J Radiol.

[36] Santilli V (2014). Clinical definition of sarcopenia. Clinical cases in mineral and bone metabolism : the official journal of the Italian Society of Osteoporosis, Mineral Metabolism, and Skeletal Diseases.

[37] Cushing KC (2018). Sarcopenia is a novel predictor of the need for rescue therapy in hospitalized ulcerative colitis patients. J Crohns Colitis.

[38] Adams DW (2017). Sarcopenia is common in overweight patients with inflammatory bowel disease and may predict need for surgery. Inflamm Bowel Dis.

[39] Higgins JPT, Chandler J, Cumpston M, Li T, Page MJ, Welch VA (2022) Cochrane handbook for systematic reviews of interventions version 6.3 (updated February 2022). Cochrane 2022. Available from www.training.cochrane.org/handbook

[40] Sterne JAC (2019). RoB 2: a revised tool for assessing risk of bias in randomised trials. BMJ.

[41] Sterne JA (2016). ROBINS-I: a tool for assessing risk of bias in non-randomised studies of interventions. BMJ.

[42] Ng V (2007). Low-intensity exercise improves quality of life in patients with Crohn’s disease. Clin J Sport Med.

[43] Robinson RJ (1998). Effect of a low-impact exercise program on bone mineral density in Crohn’s disease: a randomized controlled trial. Gastroenterology.

[44] Loudon CP (1999). The effects of physical exercise on patients with Crohn’s disease. Am J Gastroenterol.

[45] Jones K (2020). Randomised clinical trial: combined impact and resistance training in adults with stable Crohn’s disease. Aliment Pharmacol Ther.

[46] Tew GA (2019). High-intensity interval training and moderate-intensity continuous training in adults with Crohn’s disease: a pilot randomised controlled trial. BMC Gastroenterol.

[47] Seeger WA (2020). Moderate endurance and muscle training is beneficial and safe in patients with quiescent or mildly active Crohn’s disease. United European Gastroenterol J.

[48] D'Incà R (1999). Effect of moderate exercise on Crohn’s disease patients in remission. Ital J Gastroenterol Hepatol.

[49] Gupta N (2006). Effect of yoga based lifestyle intervention on state and trait anxiety. Indian J Physiol Pharmacol.

[50] Benazzato L (2006). Effect of moderate acute aerobic exercise in patients with Crohn’s disease in clinical remission. Dig Liver Dis.

[51] Cramer H (2017). Randomised clinical trial: yoga vs written self-care advice for ulcerative colitis. Aliment Pharmacol Ther.

[52] Elsenbruch S (2005). Effects of mind-body therapy on quality of life and neuroendocrine and cellular immune functions in patients with ulcerative colitis. Psychother Psychosom.

[53] Langhorst J (2007). Effects of a comprehensive lifestyle modification program on quality-of-life in patients with ulcerative colitis: a twelve-month follow-up. Scand J Gastroenterol.

[54] Cronin O (2019). Moderate-intensity aerobic and resistance exercise is safe and favorably influences body composition in patients with quiescent Inflammatory Bowel Disease: a randomized controlled cross-over trial. BMC Gastroenterol.

[55] Klare P (2015). The impact of a ten-week physical exercise program on health-related quality of life in patients with inflammatory bowel disease: a prospective randomized controlled trial. Digestion.

[56] de Souza Tajiri GJ, de Castro CLN, Zaltman C (2014) Progressive resistance training improves muscle strength in women with inflammatory bowel disease and quadriceps weakness. J Crohn Colit 8(12):1749–1750 10.1016/j.crohns.2014.09.001 25239575

[57] van Erp LW (2021). Improvement of fatigue and quality of life in patients with quiescent inflammatory bowel disease following a personalized exercise program. Dig Dis Sci.

[58] Gerbarg PL (2015). The effect of breathing, movement, and meditation on psychological and physical symptoms and inflammatory biomarkers in inflammatory bowel disease: a randomized controlled trial. Inflamm Bowel Dis.

[59] McNelly AS (2016). The effect of increasing physical activity and/or omega-3 supplementation on fatigue in inflammatory bowel disease. Gastrointest Nurs.

[60] Lamers CR (2021). Repeated prolonged moderate-intensity walking exercise does not appear to have harmful effects on inflammatory markers in patients with inflammatory bowel disease. Scand J Gastroenterol.

[61] Sharma P (2015). Effect of yoga-based intervention in patients with inflammatory bowel disease. Int J Yoga Therap.

[62] Spijkerman R (2021). Refractory neutrophils and monocytes in patients with inflammatory bowel disease after repeated bouts of prolonged exercise. Cytometry B Clin Cytom.

[63] Hassid B (2016). Effect of intense exercise on inflammatory bowel disease activity: 686. Am J Gastroenterol.

[64] Fagan G, Schultz M, Osborne H (2019) Sa1877 – individualised, unsupervised exercise program achieves high levels of compliance and improvements in patient reported outcomes - a prospective cohort study in patients with IBD. Gastroenterology 156(6, Supplement 1):S-438

[65] Mahida YR (1990). Plasma and tissue interleukin-2 receptor levels in inflammatory bowel disease. Clin Exp Immunol.

[66] Sitaraman S (2004). Colonic leptin: source of a novel proinflammatory cytokine involved in IBD. Faseb j.

[67] Kane H, Lynch L (2019). Innate immune control of adipose tissue homeostasis. Trends Immunol.

[68] Nic Suibhne T (2013). High prevalence of overweight and obesity in adults with Crohn’s disease: associations with disease and lifestyle factors. J Crohns Colitis.

[69] Erhayiem B (2011). Ratio of visceral to subcutaneous fat area is a biomarker of complicated Crohn's disease. Clin Gastroenterol Hepatol.

[70] Holt DQ (2017). Visceral adiposity predicts post-operative Crohn’s disease recurrence. Aliment Pharmacol Ther.

[71] Jones K (2022). Effects of structured exercise programmes on physiological and psychological outcomes in adults with inflammatory bowel disease (IBD): a systematic review and meta-analysis. PLoS ONE.

[72] Langhorst J (2016). Faecal lactoferrin, calprotectin, PMN-elastase, CRP, and white blood cell count as indicators for mucosal healing and clinical course of disease in patients with mild to moderate ulcerative colitis: post hoc analysis of a prospective clinical trial. J Crohns Colitis.

[73] Jacobs I (2021). Role of eosinophils in intestinal inflammation and fibrosis in inflammatory bowel disease: an overlooked villain?. Front Immunol.

[74] Canavese G (2017). Eosinophilia - associated basal plasmacytosis: an early and sensitive histologic feature of inflammatory bowel disease. APMIS.

[75] Champion K (2013). Endotoxin neutralization as a biomonitor for inflammatory bowel disease. PLoS ONE.

[76] Mc Gettigan N, O'Toole A, Boland K (2022) Role of exercise in preventing and restoring gut dysbiosis in patients with inflammatory bowel disease: a letter to the editor. World J Gastroenterol 28(8):878–880 10.3748/wjg.v28.i8.878 PMC8900572 35317102

[77] Quiroga R (2020). Exercise training modulates the gut microbiota profile and impairs inflammatory signaling pathways in obese children. Exp Mol Med.

[78] Morita E et al (2019) Aerobic exercise training with brisk walking increases intestinal bacteroides in healthy elderly women. Nutrients 11(4) 10.3390/nu11040868 PMC6520866 30999699

