# Supplementary material for: A systematic review of the effect of structured exercise on inflammation and body composition in inflammatory bowel disease
Source: Int J Colorectal Dis. 2023 May 25;38(1):143. doi: 10.1007/s00384-023-04437-2 (PMC10212817; doi:10.1007/s00384-023-04437-2)
Supplement: Supplementary file 1 — Supplementary file1 (DOCX 1005 KB) [file 384_2023_4437_MOESM1_ESM.docx]

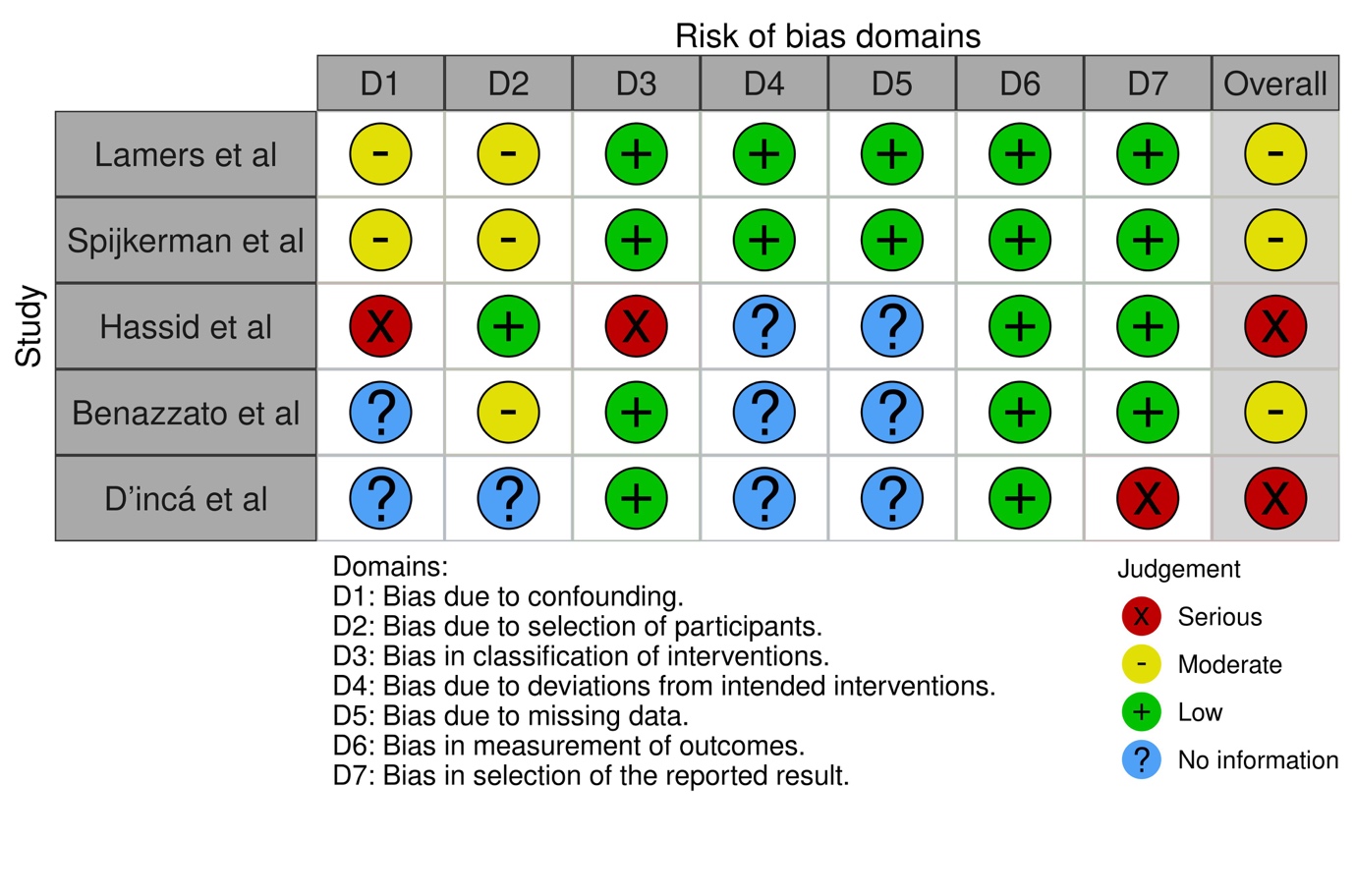


**Figure 1. Graph of bias for observational studies inflammatory response to exercise.**


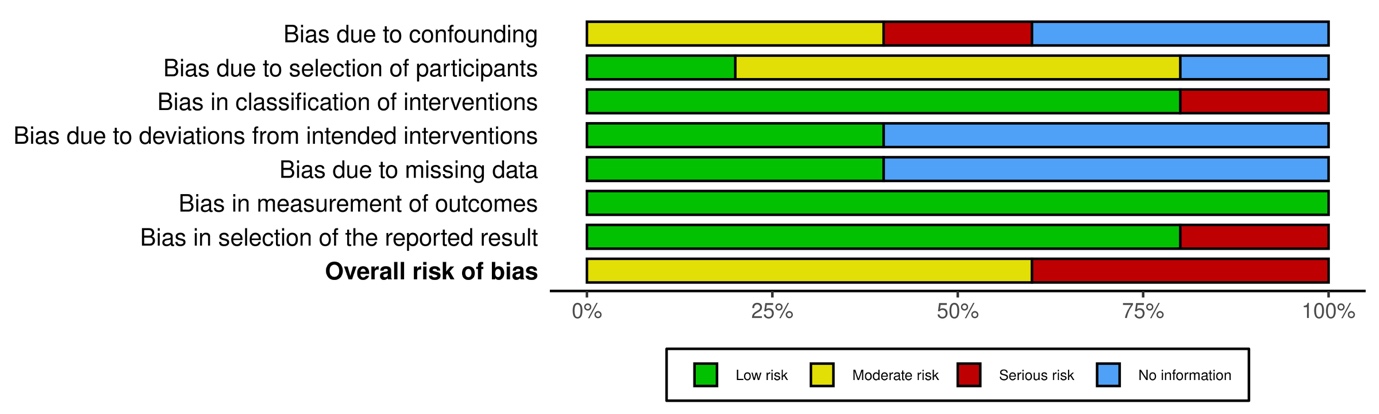


**Figure 2. Summary plot for observational studies inflammatory response to exercise.**


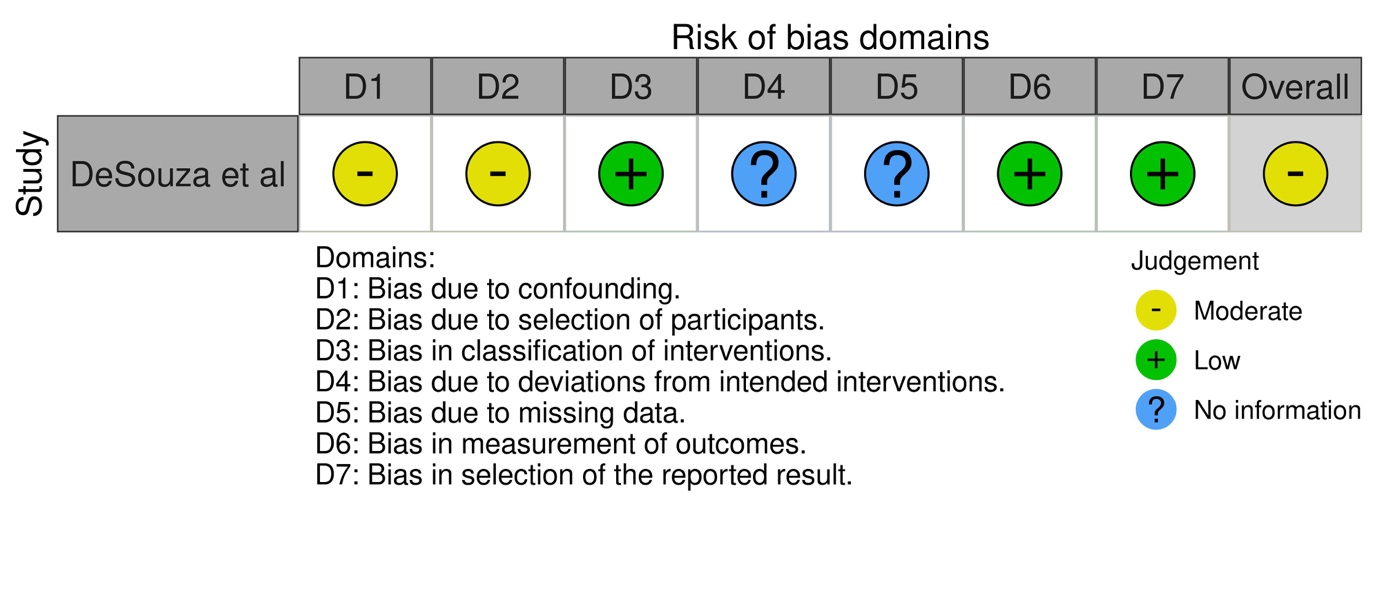


**Figure 3. Graph of bias for observational studies of body composition**

**
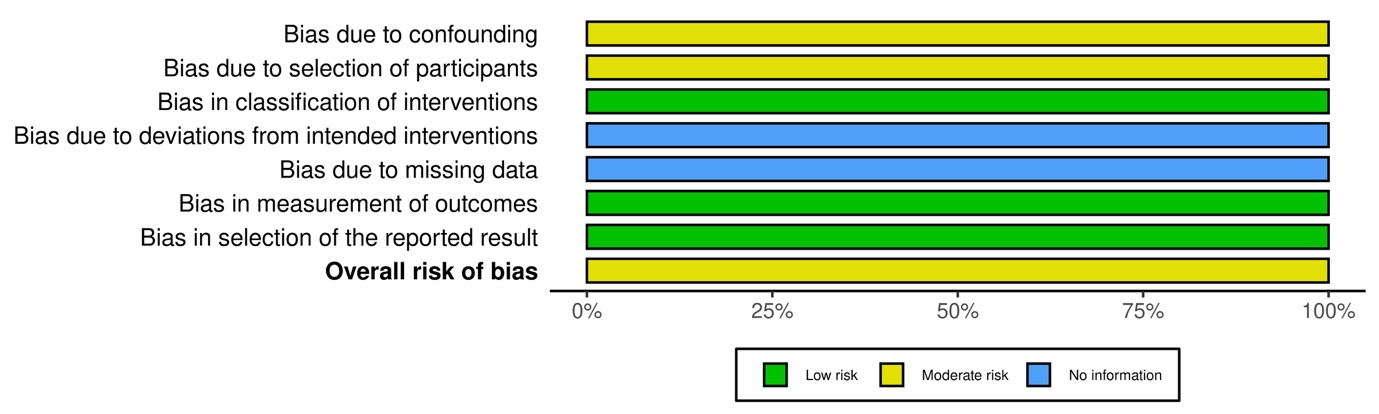
**

**Figure 4. Summary plot** **for observational studies of body composition**

**
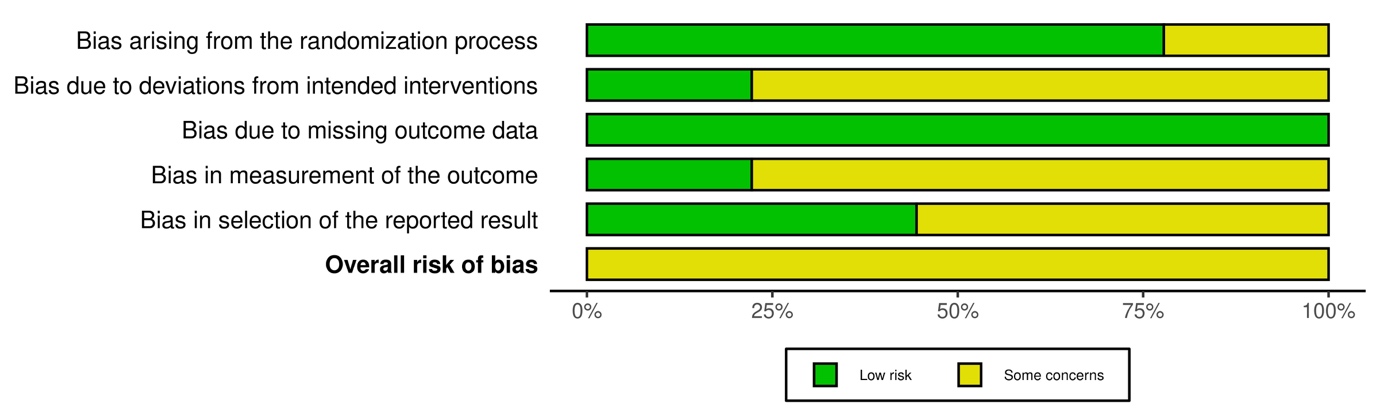
**

**Figure 5. Summary plot for inflammatory response in RCTs**

**
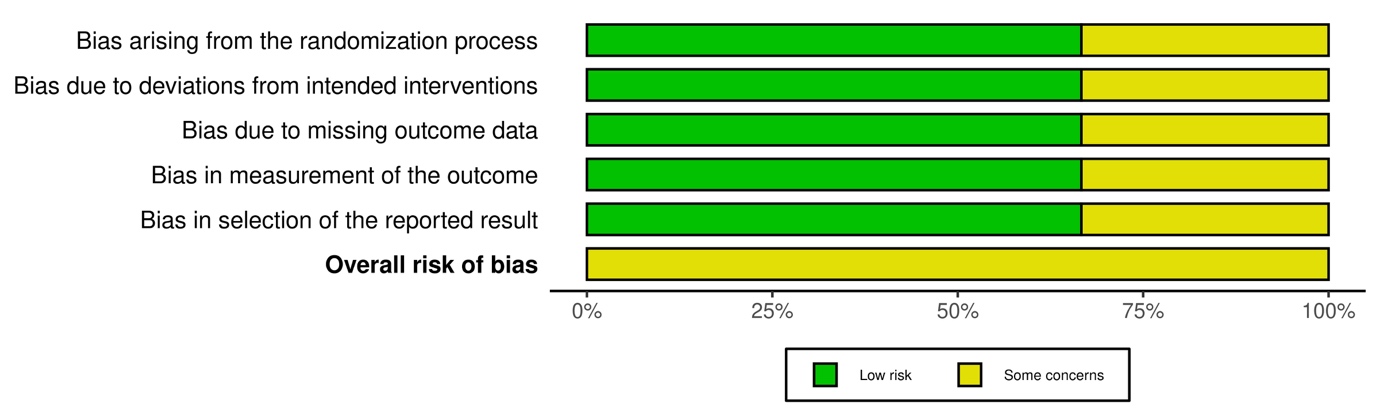
**

**Figure 6. Summary plot for body composition in RCTs**
